# LONG-DISTANCE CAREGIVERS’ INFORMAL CARE NETWORKS

**DOI:** 10.1093/geroni/igz038.2059

**Published:** 2019-11-08

**Authors:** Danielle Jimenez, Francesca Falzarano, Amy Horowitz, Verena Cimarolli, Jillian Minahan

**Affiliations:** 1 Fordham University, New York, New York, United States; 2 Fordham University, Bronx, New York, United States; 3 LeadingAge LTSS Center @UMass Boston, Washington, District of Columbia, United States

## Abstract

The purpose of this study (N=304) was to examine the characteristics of LDCs’ informal caregiver (IC) network (Co-caregivers [Co-CG], other informal helpers) providing assistance to the care recipient (CR), and factors associated with more help received from ICs. The majority of LDCs in the sample reported working with at least one IC (81.9%) indicating the existence of a secondary care network. LDCs and Co-CGs were often siblings in comparison to other informal helpers that were more likely to be the CR’s friend. Results also show that CRs with children, living in the community, receiving no formal services, and lower levels of cognitive impairment receive more hours of help from ICs. In addition, more hours of help by ICs were associated with LDCs’ having higher scores of depression and anxiety, spending more hours per month helping the CR, and more frequent contact with CR. These seemingly discrepant findings are discussed.

